# A Prospective Cohort Study Comparing Microscopy and Culture in the Diagnosis of Superficial Fungal Skin Infections

**DOI:** 10.3390/medsci13040247

**Published:** 2025-10-29

**Authors:** Amelia Yuting Monteiro, Hui Mei Cheng, Larissa Lim, Jiun Yit Pan, Kun Liang, Hong Liang Tey

**Affiliations:** 1 Duke-NUS Medical School, Singapore 169857, Singapore; 2 National Skin Centre, Singapore 308205, Singaporeteyhongliang@ntu.edu.sg (H.L.T.); 3 A*Star Skin Research Labs (A*SRL), Singapore 138648, Singaporekun.liang@ntu.edu.sg (K.L.); 4 Lee Kong Chian School of Medicine, Nanyang Technological University, Singapore 308232, Singapore; 5 Skin Research Institute of Singapore, Singapore 308232, Singapore

**Keywords:** fungal, infection, treatment, microscopy, culture, dermatophyte

## Abstract

Superficial fungal skin infections are common but often misdiagnosed, which may result in inappropriate treatment and the worsening of symptoms. An accurate and timely diagnosis is essential to differentiate these infections from similar conditions such as secondary syphilis, annular psoriasis, and pityriasis rosea. This single-centre prospective cohort study at the National Skin Centre, Singapore, evaluated the diagnostic agreement between direct microscopy and fungal culture. Between August and December 2022, 268 skin scrape samples were collected from 149 patients with suspected fungal infections. Microscopy identified 67 (25.0%) positives, while fungal culture detected 42 (16.7%) positives. Among the 252 samples tested with both methods, 213 (84.5%) showed concordant results (κ = 0.487, *p* < 0.0001), a finding that indicates moderate agreement. The most commonly cultured organisms were *Trichophyton rubrum* and *T. mentagrophytes*. Our findings suggest that both microscopy and fungal culture may be performed to prevent true-positive cases from being missed. However, in cases where cost is a concern, microscopy can be selected as an initial diagnostic tool. Should microscopy be negative in cases with high clinical suspicion for fungal infection or when empirical treatment fails, culture remains a valuable follow-up test. These findings support a stepwise diagnostic approach—using microscopy first, then followed by culture when necessary—to improve diagnostic accuracy while enabling timely treatment.

## 1. Introduction

Superficial fungal skin infections represent a global health concern due to their high prevalence and rising incidence [1,2]. These infections, characterized by itchy and scaly skin, can cause significant distress to patients [3]. However, fungal skin infections are often clinically challenging to distinguish from other non-fungal skin diseases such as secondary syphilis, annular psoriasis, and pityriasis rosea [4]. Therefore, investigations are often required for differentiation prior to initiating treatment.

Common diagnostic methods include direct microscopic examination (“microscopy”) and/or fungal culture (“culture”) of skin specimens, investigations which are obtained via skin scrapings of the affected skin. These two diagnostic tests can be performed singly or in conjunction at the clinician’s discretion. In general, the direct microscopy of skin specimens to visualize the presence of fungal elements is higher in sensitivity, less costly, and allows for rapid diagnosis [5,6] compared to culturing. The latter takes an estimated 6 weeks to obtain results, a timeframe that leads to a lag in diagnosis but is more specific, as many specimens determined to be positive by microscopy fail to grow in culture [7]. However, microscopy—without a fungal culture—is limited in its ability to completely identify all fungal species, likely from the higher limit of detection for microscopy. Given a worldwide trend suggesting emerging fungal resistance patterns [8], the ability to leverage both the sensitivity of fungal culture and the rapid turnaround time for fungal microscopy becomes increasingly important. Notably, frequently implicated species that cause superficial fungal skin infections in Singapore were *T. rubrum* (58%), followed by *Epidermophyton floccosum* (14%) and *T. mentagrophytes* (10%) [9]. In vitro evaluation revealed that the majority of the isolates were sensitive to the three drugs tested (griseofulvin, ketoconazole, and itraconazole [9]. However, since epidemiological patterns vary across geographical regions, climate, and changes over time, an epidemiological update is necessary in Singapore’s context.

Despite being common investigations for superficial fungal skin infections, the concordance between microscopy and fungal culture remains unclear. The current literature reports Cohen’s Kappa coefficient (κ) as low as 0.094, a value that indicates poor agreement (*p* value = 0.047) between culture and microscopy in the diagnosis of superficial dermatophytes [10]. Here, we conducted a prospective cohort study to understand the concordance between fungal microscopy and culture in guiding the diagnosis of superficial fungal infections. Additionally, we determined the types of fungal species isolated from positive culture tests in Singapore.

## 2. Materials and Methods

This was a single-centre, prospective cohort study with specimen collection conducted from August to December 2022. Approval was obtained from the institution’s ethics board (2022/00341; date of approval: 18 August 2022). Eligible participants were patients aged ≥21 years whose clinicians suspected a superficial fungal infection and had not used oral or topical antifungal agents within the preceding two weeks. Informed consent was obtained from all subjects involved in the study.

Eligible participants first underwent skin scraping of the affected areas to obtain a sufficient amount of skin sample. Skin scraping was performed by a professional clinical technician, during which a plastic scalpel was used to scrape the surface of affected skin to obtain skin samples. The skin samples were then collected in a sterile bottle to be subject to direct microscopy and culture, performed according to National Skin Centre’s laboratory guidelines, and were performed by trained laboratory staff.

### 2.1. Investigation Methods

#### 2.1.1. Microscopy

**Figure 1:**
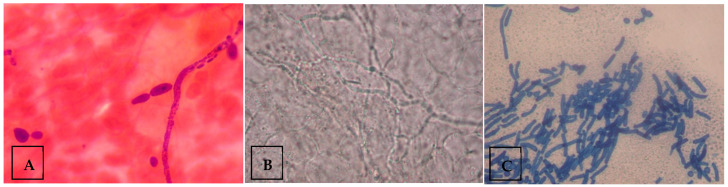
Examples of fungal morphology visualized under the microscope. (**A**) Microscopic image depicting budding yeast cells with pseudohyphae, indicating *Candida* species. (**B**) Microscopic image depicting mycelium, indicating dermatophytes. (**C**) Microscopic image depicting spores and hyphae, indicating *Malassezia* species. Figure contributed by author.

For microscopy samples, skin samples were placed onto a clean glass slide with 15% potassium hydroxide (KOH) solution and mounted with a coverslip. The slide was then placed on a warm heater of 50 °C for five minutes to soften the material. The preparation was then examined under low-power magnification (10×). Fungal elements or spores, if any, were then confirmed using a higher-power magnification (40×). The visualization of fungal morphology indicated a positive sample, while the absence of it indicated a negative sample. The presence of budding yeast cells with pseudohyphae on microscopy was considered diagnostic of *Candida* species (Figure 1A), whereas visualization of mycelia indicated dermatophytes (Figure 1B), and the presence of both spores and hyphae suggested *Malassezia* species (Figure 1C). Samples demonstrating spores and hyphae, consistent with *Malassezia furfur*, were excluded from culture, as routine laboratory protocols do not include culturing for *M. furfur*.

#### 2.1.2. Culture

Two different culture mediums were used: (1) Sabouraud Dextrose Agar with Chloramphenicol and (2) Sabouraud Dextrose Agar with Chloramphenicol and Cycloheximide. With a sterile inoculating needle, equal amounts of specimen were inoculated onto both culture plates. Culture plates were incubated at 28 °C and examined twice a week for a minimum of 21 days for any fungal colonies. Fungal colonies, if present, were subjected to both macroscopic examination and microscopic examination for identification of fungal species. The growth of fungal colonies on culture plates would indicate a positive sample, while the absence would indicate a negative sample.

### 2.2. Statistical Analysis and Sample Size

For statistical analysis, our power calculations demonstrate that a sample size of 266 specimens would result in a two-sided 95% confidence interval with a width of 0.25 for the value of κappa at 0.400 and the expected proportion of agreement at 0.75 for both raters [10,11,12]. The SD(κ) was estimated using Cohen’s large-sample formula [13], and the percentage agreement and Cohen’s κappa were calculated. Statistical analysis was performed using SPSS version 24.

## 3. Results

**Figure 2:**
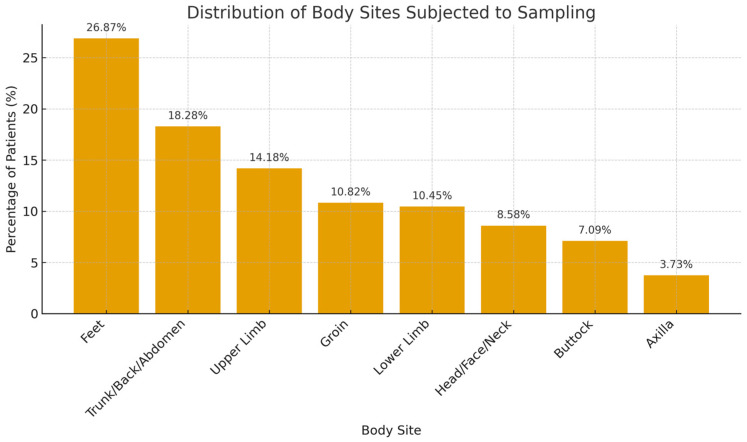
Distribution of body sites sampled for fungal testing.

A total of 268 samples were collected from 149 patients who underwent fungal scraping between September and December 2022. The age range was between 21 and 83 years (SD = 17.0). Among the 149 patients, 77 (51.7%) had two or more areas from different sites of the body subjected to skin sampling (Table 1). A total of 72 (26.9%) samples were taken from the feet, 49 (18.28%) from the trunk, back, and abdomen, 38 (14.18%) from the upper limbs, 29 (10.82%) from the groin, 28 (10.45%) from the lower limbs, 23 (8.58%) from the head, face, and neck, 19 (7.09%) taken from the buttock, and 10 (3.73%) from the axilla (Figure 2).

### 3.1. Results from Microscopy and Culture

Microscopy was first performed on all 268 samples. A total of 201 (75.0%) samples tested negative, while 67 (25.0%) tested positive (Table 2). Microscopic visualization of fungal morphology in positive samples showed 46 (68.7%) samples with mycelium (indicating dermatophytes), 16 (23.9%) with spores and hyphae (indicating *Malassezia* species), and 5 (7.46%) budding yeast cells with pseudohyphae (indicating *Candida* species) (Table 2). Sixteen specimens that had spores and hyphae visualized under microscopy (indicative of *Malassezia furfur*) were excluded from the culture as our laboratory does not routinely culture for *M. furfur.* Fungal culture was then performed on the remaining 252 specimens. For fungal culture specimens, 210 (83.3%) were negative, while 42 (16.7%) were positive for fungus (Table 1). The most common species isolated in positive fungal cultures are *Trichophyton rubrum* (52.4%), *Trichophyton mentagrophytes variant interdigitale* (28.6%), *Candida albicans* (11.9%), *Candida parapsilosis* (4.7%), and *Fusarium* spp. (2.4%) (Table 2).

### 3.2. Comparison Between Results of Microscopy and Culture

For 252 cases that were subjected to both microscopy and culture, 213 out of 252 (84.5%) samples revealed concordant results from both tests—27 samples (10.7%) were both positive and 186 (73.8%) were both negative (Table 3). This resulted in an 84.5% overall agreement between microscopy and culture, with a Cohen’s Kappa coefficient of 0.487 (*p*-value < 0.0001), values that suggest moderate agreement. However, 15 samples, which were microscopy-negative, turned out to be culture-positive, while 24 microscopy-positive samples turned out to be culture-negative (Table 3).

## 4. Discussion

Superficial fungal skin infections represent a global health concern due to their high prevalence and rising incidence [1,2]. An analysis of fungal skin disease trends in 2017 in 195 countries worldwide found that the prevalence of fungal skin disease in both sexes was approximately 750 million, and the global age-standardized disability-adjusted life years (DALYs) per 100,000 people with fungal skin disease was 54.86 for both sexes, 56.48 for men, and 53.17 for women [14]. Locally, a total of 12,903 cases of superficial fungal infections were seen at the National Skin Centre, Singapore, from 1999 to 2003, with a rising trend observed over time [1].

Fungal skin infections can cause chronic pruritus and discomfort if left untreated. These infections can commonly be mistaken for other types of skin disease such as secondary syphilis, annular psoriasis, and pityriasis rosea [4], and an accurate and prompt diagnosis is needed to initiate appropriate treatment. It is therefore crucial to have readily available diagnostic investigations for fungal identification clinically. Direct microscopy of skin specimens remains a rapid, inexpensive, and sensitive diagnostic method, though it does not allow for fungal speciation—an advantage offered by fungal culture.

In our study, the feet were the most commonly affected site (26.9%), and *Trichophyton rubrum* was the predominant causative organism. Ideally, antifungal treatment should be selected based on the causative organism isolated for efficacy and appropriateness. For instance, terbinafine demonstrates fungicidal activity against dermatophytes and is generally more effective and better tolerated than griseofulvin [15]. Conversely, azole antifungal agents such as fluconazole and itraconazole have been widely used to treat superficial fungal infections caused by dermatophytes and have been associated with resistance development [16].

We found an overall agreement of 84.5% between microscopy and fungal culture, with a Cohen’s Kappa coefficient of 0.487 (*p* < 0.0001), indicating moderate agreement. Fifteen microscopy-negative samples were culture-positive, while 24 microscopy-positive samples were culture-negative. This highlights the risk of missed diagnoses when relying on a single diagnostic modality.

As such, the authors propose that both tests should ideally be performed to maximize the detection of true-positive superficial fungal infections. However, in cases requiring a quicker turnaround time or with cost considerations, we suggest that microscopy be performed as an initial investigation since it is cheaper and can provide a quicker diagnosis for the timely initiation of treatment in positive cases. Treatment should be withheld until the microscopy results are available. Should microscopy results be negative and the clinical suspicion for fungal infection remain low, the physician should consider an alternative diagnosis and treatment, with adequate follow-up to evaluate for treatment efficacy.

Conversely, in microscopy-negative cases with atypical presentations, recurrent or chronic infections, poor response to empirical therapy, or where antifungal resistance is suspected, cultures should then be prioritized. The clinician should proceed with a fungal culture and start empiric antifungal therapy, with a return appointment in 4–6 weeks for the patient to review the response to therapy and fungal culture results.

This study has several limitations. First, it was conducted at a single centre, a limitation which may affect the generalizability of the findings to other settings with different patient populations and fungal epidemiology. We do note that Singapore is a multi-ethnic and multi-cultural tropical country with a high prevalence of fungal skin infections, a feature that helps mitigate concerns regarding population homogeneity and/or clinical relevance. Second, the interpretation of microscopy results is inherently operator dependent. Here, we did not assess inter-observer variability, a factor which could influence diagnostic accuracy. Future studies would include a multi-centre design and implement standardized training protocols for evaluating the inter-observer agreement for fungal microscopy. Lastly, a limitation of this study is the lack of data on patient comorbidities, such as diabetes mellitus or immunosuppressive states, which may influence susceptibility to fungal infections. Future prospective studies incorporating detailed clinical and comorbidity data would allow for a more comprehensive analysis of host factors influencing infection risk.

## 5. Conclusions

In conclusion, we found a moderate 84.5% agreement between fungal microscopy and culture results. Depending on the duration of results that are needed and if cost is an issue, clinicians can better decide whether to perform microscopy alone or concurrently perform a fungal culture at the first patient consultation.

## Figures and Tables

**Table 1:** Number and percentage of patients who had 1 to 6 body sites subjected to sampling, and the types of body sites subjected to sampling.

Number of Body Sites Subjected to Sampling	Number of Patients (%)
1	72 (48.32)
2	51 (34.23)
3	16 (10.74)
4	5 (3.36)
5	4 (2.68)
6	1 (0.67)
Total	149 (100)

**Table 2:** Results of skin samples that underwent microscopy and culture.

**Microscopy Result**	**Number of Samples**	**Percentage**	**Fungal Morphology Observed**
Negative	201	75.00%	No Fungal morphology seen
Positive	67	25.00%	Budding yeast: 5 (7.46%); spores and hyphae: 16 (23.88%); and mycelium: 46 (68.66%)
**Total**	**268**	**100.00%**	
**Culture Result**	**Number of Samples**	**Percentage**	**Fungal Species Isolated in Positive Cultures**
Negative	210	83.33%	No Fungal species isolated
Positive	42	16.67%	*Trichophyton rubrum*: 22 (52.38%) *Trichophyton mentagrophytes variant interdigitale*: 12 (28.57%) *Candida albicans*: 5 (11.90%) *Candida parapsilosis*: 2 (4.76%) *Fusarium spp*.: 1 (2.38%)
**Total**	**252**	**100%**	

**Table 3:** Comparison table showing number of cases with positive and negative results in both microscopy and culture.

		Microscopy		
		Positive	Negative	Total
**Culture**	**Positive**	27	15	42
	**Negative**	24	186	210
	**Total**	51	201	252

## Data Availability

The original contributions presented in this study are included in the article. Further inquiries can be directed to the corresponding author.
